# *De Novo* Transcriptome Assembly (NGS) of *Curcuma longa* L. Rhizome Reveals Novel Transcripts Related to Anticancer and Antimalarial Terpenoids

**DOI:** 10.1371/journal.pone.0056217

**Published:** 2013-02-28

**Authors:** Ramasamy S. Annadurai, Ramprasad Neethiraj, Vasanthan Jayakumar, Anand C. Damodaran, Sudha Narayana Rao, Mohan A. V. S. K. Katta, Sreeja Gopinathan, Santosh Prasad Sarma, Vanitha Senthilkumar, Vidya Niranjan, Ashok Gopinath, Raja C. Mugasimangalam

**Affiliations:** Research and Development Unit, Genotypic Technology Private Limited, Bangalore, Karnataka, India; The University of Texas M. D. Anderson Cancer Center, United States of America

## Abstract

Herbal remedies are increasingly being recognised in recent years as alternative medicine for a number of diseases including cancer. *Curcuma longa* L., commonly known as turmeric is used as a culinary spice in India and in many Asian countries has been attributed to lower incidences of gastrointestinal cancers. Curcumin, a secondary metabolite isolated from the rhizomes of this plant has been shown to have significant anticancer properties, in addition to antimalarial and antioxidant effects. We sequenced the transcriptome of the rhizome of the 3 varieties of *Curcuma longa* L. using Illumina reversible dye terminator sequencing followed by *de novo* transcriptome assembly. Multiple databases were used to obtain a comprehensive annotation and the transcripts were functionally classified using GO, KOG and PlantCyc. Special emphasis was given for annotating the secondary metabolite pathways and terpenoid biosynthesis pathways. We report for the first time, the presence of transcripts related to biosynthetic pathways of several anti-cancer compounds like taxol, curcumin, and vinblastine in addition to anti-malarial compounds like artemisinin and acridone alkaloids, emphasizing turmeric's importance as a highly potent phytochemical. Our data not only provides molecular signatures for several terpenoids but also a comprehensive molecular resource for facilitating deeper insights into the transcriptome of *C. longa*.

## Introduction

*Curcuma longa*, commonly known as turmeric, is a rhizomatous small perennial plant from the ginger family (Zingiberaceae). Turmeric has been used as a colouring and flavouring additive in day to day cooking in India and many east asian countries for centuries and also used as a household remedy for many ailments. Turmeric at alkaline PH turns bright red and it is widely used as Vermilion, also known as kumkum, an important part of Hindu religious ceremonies. Over the last few decades, turmeric has gained global recognition for its medicinal importance after many studies that were conducted to understand its medicinal properties, yielded exciting results. The primary active constituent of turmeric is an important secondary metabolite namely, curcumin. It's role as an antimalarial [1], anti-inflammatory [2], [3] and antitumor [3] compound has been well appreciated worldwide and it has also been known to modulate lipid metabolism, which has been implicated in obesity [4]. In addition, curcumin has also been used in clinical trials to treat Azheimer's [5].

Turmeric oil/oleoresin, extracts which contain curcuminoids and essential oils are used for flavouring and colouring. It has been shown that turmeric oleoresin has hypoglycemic [6], anti-amyloidogenic [7], paracitidal [8], antimicrobial [9] and larvicidal [10] effects. Apart from curcumin there are many compounds in turmeric oil that contribute to its above mentioned properties. Nonetheless, global efforts in turmeric have been concentrated on studying and modifying curcuminoid biosythetic pathway and a thorough study of transcriptome is so far not attempted. Such studies might shed light into the functional genes and aid to understand the diverse pathways involved in phytomedical attributes of turmeric.

In recent years a number of non-model plants have been sequenced using various Next Generation Sequencing (NGS) platforms [11]–[22]. Despite turmeric's growing medicinal and economic importance, a comprehensive transcriptome level investigation is lacking. In this study an attempt has been made to analyse and annotate *C. longa* transcriptome from the rhizomes of three popularly cultivated cultivars in south India by assembling short paired-end Illumina reads. Cultivar Nattu (traditional) yeilds small rhizomes, cultivar Erode is widely grown commercial variety with larger rhizomes and cultivars Mysore requires higher irrigation with lower maturation time. Expression studies were conducted to observe differences across the three cultivars. The transcriptome will serve as an invaluable genomic reference to further our knowledge about turmeric at a molecular level.

## Results

### Sequence Quality Control

A total of 20,519,880×2 (72 base), 30,342,598×2 (73 base), 37,193,403×2 (100 base) raw reads were generated from Illumina GAIIx sequencer, accounting for approximately 2.9 Gb, 4.4 Gb and 7.4 Gb of sequence data, for cultivars Nattu, Erode and Mysore respectively. The raw paired-end sequence data in FASTQ format was deposited in the National Centre for Biotechnology Information's (NCBI) Short Read Archive (SRA) database under the accession number SRA052613. Raw reads were subjected to quality control (SeqQC). High quality (>Q20) bases were more than 90% in both the forward and the reverse (paired-end) reads (Table 1). After removing the adapter and low quality sequences from the raw data, 34,924,986, 48,755,296 and 63,574,950 high quality reads were retained for cultivars A, B and C respectively. These high quality, processed paired-end reads were used for further analysis.

**Table 1:** Summary of RNA-Seq.

	Cultivar Nattu	Cultivar Erode	Cultivar Mysore
Number of raw reads*	41,039,760	60,685,196	74,386,806
Read length	72	73	100
Number of High Quality (HQ) bases**	2,786,398,656	4,124,692,295	6,787,408,561
Percentage of HQ bases	94.3	93.1	91.2
Reads after trimming adapters and low quality bases	34,924,986	48,755,296	63,574,950
Number of bases in trimmed reads	2,496,109,122	3,525,038,126	6,085,481,848
Mean trimmed read length	71.47	72.30	95.72
Median trimmed read length	72	73	100

* Reads = Read1+Read2.

** Bases with >20 Phred score.

### Transcriptome Assembly and Clustering

**Figure 1:**
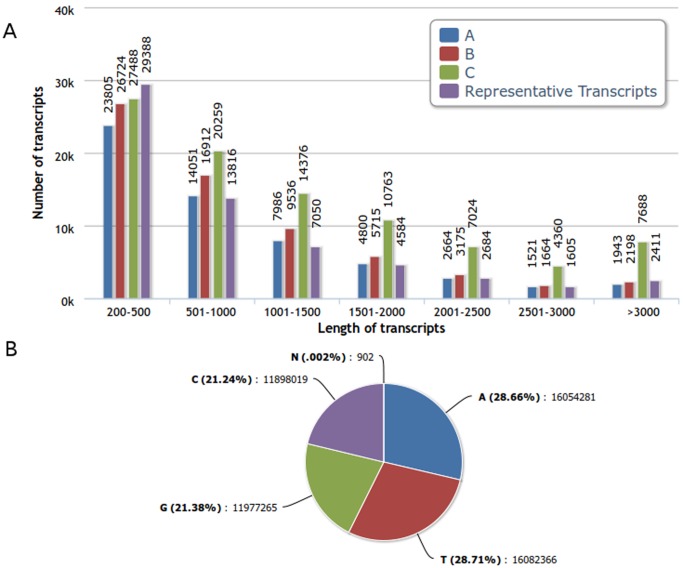
Transcript assembly statistics. A) Length of the assembled transcripts vs. Number of transcripts B) ATGC composition of the RTs.

Filtered reads were assembled into contigs using Velvet at a hash length of 45, which generated 137,148, 91,995 and 203,400 contigs for cultivars Nattu, Erode and Mysore respectively. Further transcriptome assembly using Oases resulted in 56,770, 65,924 and 91,958 transcripts. Figure 1A shows the transcript length distribution ranging from 200 bases to more than 3000 bases. We pooled and further assembled the individual assemblies of the three cultivars to create a reference sequence for comparative analysis. Representative transcripts (RTs) obtained after clustering using CD-HIT contained 9,568, 13,679 and 38,300 transcripts from cultivars Nattu, Erode and Mysore respectively. Clustering resulted in 61,538 RTs.

The percentage of Ns in the assembly were found to be minimal: approximately 0.001% for cultivars Nattu and Erode 0.004% for cultivar Mysore and 0.002% for RTs. Total length of RTs was found to be approximately 56Mb and the mean transcript length was 910 bases (Table 2). RTs were observed to be marginally AT rich, with 57.37% AT content (Figure 1B). The RTs can be accessed at TSA within the accession number range JW751789-JW813326.

**Table 2:** Assembly summary of cultivar A, cultivar B, cultivar C, ArREST ESTs and representative transcripts.

	Cultivar Nattu	Cultivar Erode	Cultivar Mysore	ArREST	Representative Transcripts
No of Transcripts	56,787	65,956	92,214	78,516	61,538
Maximum transcript length	15,271	11,938	15,293	6,639	15,293
Minimum transcript length	200	200	200	100	200
Total transcript length (bases)	53,751,599	62,409,692	120,256,594	21,494,172	56,012,833
Number of Ns	537	839	4,775	19,766	902
Mean transcript length	946.55	946.23	1304.10	273.755	910.22
N50	1,466	1,448	1,995	467	1,515

### BLAST Against *C. longa* Nucleotide Sequences and ESTs from ArREST

**Figure 2:**
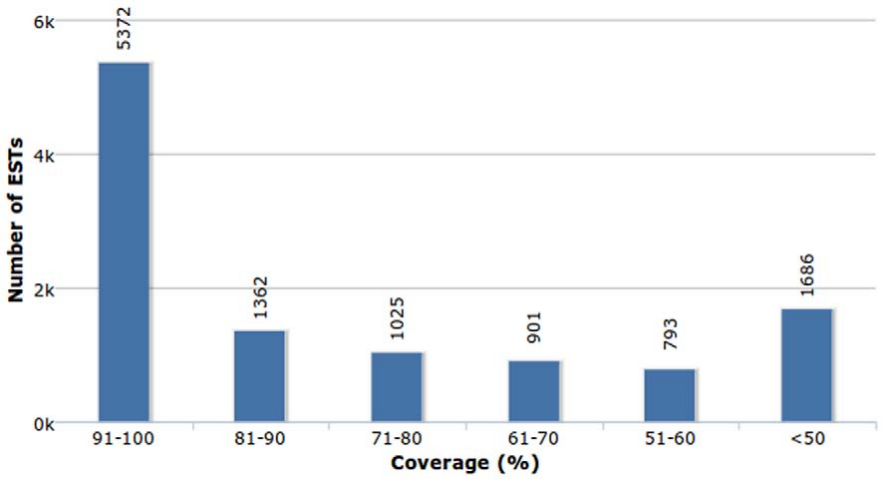
Coverage distribution of NCBI *C. longa* ESTs matched against representative transcripts using BLAST.

Sequence similarity search between RTs and GenBank's *C. longa* ESTs showed that 9,307 (15.1%) RTs were similar to 11,139 (86.8%) *C. longa* ESTs at an E-value cut-off of e-5 (<0.00001). Of these, 11,115 sequences matched with a sequence identity greater than 80% while the remaining sequences matched with an identity above 70%. A vast majority of the ESTs (5,372) were observed to align with more than 90% coverage (Figure 2). This search also revealed the presence of curcumin synthase in the transcriptome (Additional file S1).

Sequence similarity search between RTs and ArREST ESTs revealed that 68,983 (87%) of the ArREST ESTs were identified in RTs. N50 value of RTs (1,515 bases) was significantly higher than that of ArREST ESTs (467 bases). We obtained a maximum contig length of 15,293 bases as opposed to 6,639 bases of ArREST ESTs. ArREST contained only 22,560 (28.7%) transcripts above 200 bases, which explains the lower mean transcript length of ArREST ESTs (273.76 bases) when compared to that of RTs (910.14 bases). Overall our dataset (RTs) represents transcripts with much longer sequences and better transcriptome coverage (Additional file S2).

### Functional Annotation

We utilized six different databases in this study to annotate *C. longa,* as there were very limited reference information available. *In toto*, 33,614 (54.6%) RTs were annotated against all six databases. Around 33% (20,436) of RTs received Swiss-Prot annotation (Additional file S3). Out of these, 679 (3.3%) were annotated as Putative proteins while 2,285 (11.2%) were annotated as proteins with probable functions. This indicates that a large proportion of proteins received a definitive annotation from Swiss-Prot. GO terms were retrieved from Swiss-Prot annotation. About 33,172 (53.9%) of RTs were annotated with TrEMBL, an automatically annotated relatively larger database when compared to Swiss-Prot but poor in information, increases the chances of annotation with a previously known protein. Annotation against PlantCyc database resulted in the annotation of 8,789 (14.3%) RTs with enzymes from 255 pathways (Additional file S4). KOG and Genbank annotation resulted in the annotation of 19,383 (31.5%) and 3,311 (5.4%) RTs respectively. We also observed that around 39% (23,992) RTs received Pfam annotations.

**Figure 3:**
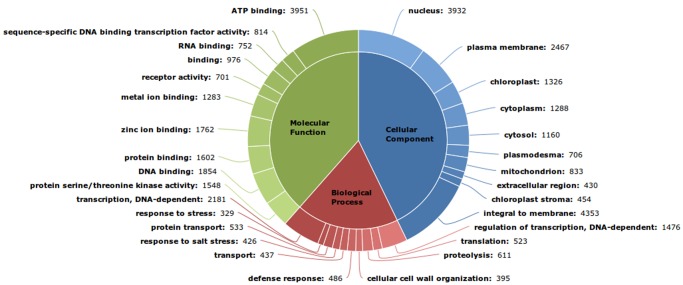
Top ten most represented GO terms in each of the three GO domains.

GO annotation showed that the annotated RTs represent genes with diverse functionalities and are involved in various metabolic pathways. We observed 26,638, 37,689, and 31,759 GO terms representing Cellular component, Molecular function and Biological process categories. In the cellular component category, the terms *integral to membrane* and *nucleus* were observed to occur most frequently, constituting 16.3% (4,353) and 14.8% (3,932) of total cellular component entries respectively. *ATP binding* and *DNA binding* were found be the most frequently occurring under molecular function category, constituting 10.5% (3,951) and 4.9% (1,854). In the biological process category *transcription,DNA-dependent* and *regulation of transcription,DNA-dependent* were observed most frequently constituting 6.9% (2,181) and 4.6% (1,476). Of transcripts with an assigned biological process term, *response to stress, defense response* and *response to salt stress* were also observed to occur more frequently, together constituting 3.9% (1,241) (Figure 3). Since, the rhizome is buried in soil it is more prone to pathogen attacks and salt stress, hence it is expected to find defense and stress related terms in high numbers. Such higher occurrences of stress related categories, also indicates the possible presence of a large number of secondary metabolites.

**Figure 4:**
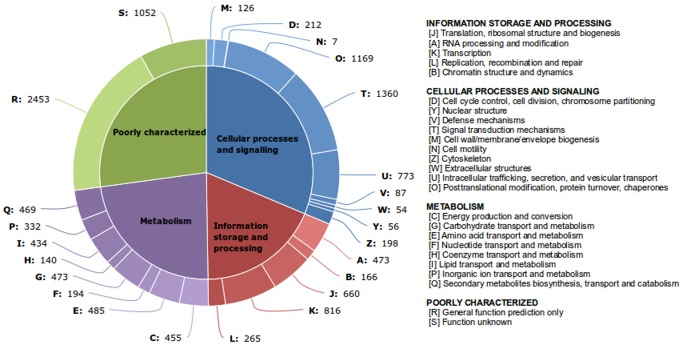
KOG Classification.

Each KOG cluster contains a protein or a group of proteins from at least 3 different eukaryotic lineages further clustered based on their function. All 25 KOG functional categories were represented in the annotation (Additional file S5). Annotation with KOG also revealed that 469 RTs, constituting 2.4% of total RTs annotated with KOG, fell in *secondary metabolites biosynthesis, transport and catabolism* category reflecting the vast repertoire of secondary metabolites present in the plant (Figure 4).

Pathway annotation revealed the presence of many important secondary metabolite pathways which synthesize compounds with diverse medicinal properties. Analysis indicated the presence of compounds with anti-cancer (taxol, matairesinol), anti-oxidant (flavonoids), antimalarial (acridone alkaloids, artemisinin), pesticidal (Benzoxazinoids) and antibiotic (hypericin) properties. The other terpenoid and flavonoid pathways represented in the transcriptome are xanthohumol, gentiodelphin, leucodelphinidin, pelargonidin, leucoperlargonidin, leucocyanidin, shisonin, syringetin, bixin, glyceollin, hesperitin and pinobanksin.

**Figure 5:**
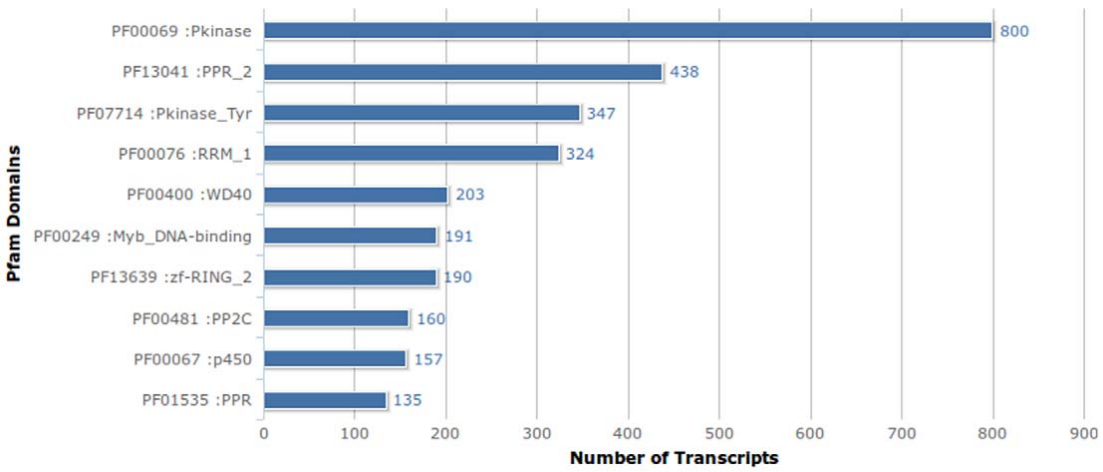
Top ten most expressed Pfam domains.

For distantly related homologs, sequence similarity search may not yield significant information, hence transcripts were searched for the presence of conserved protein domains (Additional file S6). Protein kinases (PF00069 and PF07714) were found to be the most abundant domains and given their involvement in a variety of cellular processes, including metabolism, transcription, cell movement, differentiation and apoptosis, their abundance is expected. Annotation revealed the presence of Myb_DNA-binding domain, which is the sixth most occurring domain. This domain is responsible for the transcription factor activity of Myb transcription factor superfamily, which plays regulatory role in plant developmental process and defense responses. Cytochrome P450 superfamily (PF00067) is a large and diverse group of enzymes involved in catalysing the oxidation of organic substrates and the presence of P450 domain as the eighth most occurring domain supports their abundance. The other domains such as PPR_2 repeat family (PF13041), RNA recognition motif (PF00076), RING finger domain (PF13639), Protein phosphatase 2C (PF00481), WD40 (PF00400), PPR (PF01535) were also found aplenty (Figure 5).

Annotations from individual databases were used in interpreting findings and a final annotation table was obtained in order to arrive at a single best annotation for each transcript. The final annotation table (Additional file S7) comprised of 15,632 (46.51%) RTs annotated with Swiss-Prot database, 2,437 (7.25%) RTs with PlantCyc database, 8,322 (24.76%) RTs with KOG proteins database, 6,829 (20.31%) RTs with TrEMBL database, 116 (0.35%) RTs with GenBank Viridiplantae nucleotide sequences and 277 (0.82%) RTs with Pfam database (Table 3). TrEMBL initially had the highest share of annotations. However, in the final annotation table, a major share of the results was distributed among the well annotated databases (Swiss-Prot and KOG).

**Table 3:** Annotation summary.

Database	Version	Transcripts	Percentage of transcripts
GenBank-NT	As of 14^th^ March 2012	116	0.35%
KOG	As of 9^th^ April 2012	8,322	24.76%
PlantCyc	Version 2.0	2,437	7.25%
Swiss-Prot	As of 21^st^ March 2012	15,632	46.51%
TrEMBL	As of 21^st^ March 2012	6,829	20.31%
Pfam	Version 26.0	277	0.82%

### Mapping, Calling Variations and Quantifying Transcripts

The reads from cultivars Nattu, Erode and Mysore were aligned back to RTs. Alignments showed that 90.40% reads from cultivar Nattu, 91.24% reads from cultivar Erode and 93.07% reads from cultivar Mysore were aligned to the RTs (Table 4). Alignment file was processed by SAMtools for variant calling. In cultivar Nattu, 1,00,920 variations were classified as homozygous SNPs while 2,93,230 variations were classified as heterozygous SNPs. In cultivar Erode, 1,01,566 variations were classified as homozygous SNPs while 3,34,622 variations were classified as heterozygous SNPs. In cultivar Mysore, 48,769 variations were classified as homozygous SNPs while 3,91,995 variations were classified as heterozygous SNPs.

**Table 4:** Alignment summary of Cultivars A, B and C.

Statistics	Cultivar Nattu	Cultivar Erode	Cultivar Mysore
Total reads	34,924,986	48,755,296	63,574,950
Reads aligned	31,572,848	44,488,764	59,171,432
%Reads aligned	90.40	91.24	93.07
Reference sequence length	56,012,833	56,012,833	56,012,833
Total reference covered	48,952,920	49,806,266	53,364,114
% Total reference covered	87.40	88.89	95.27

**Figure 6:**
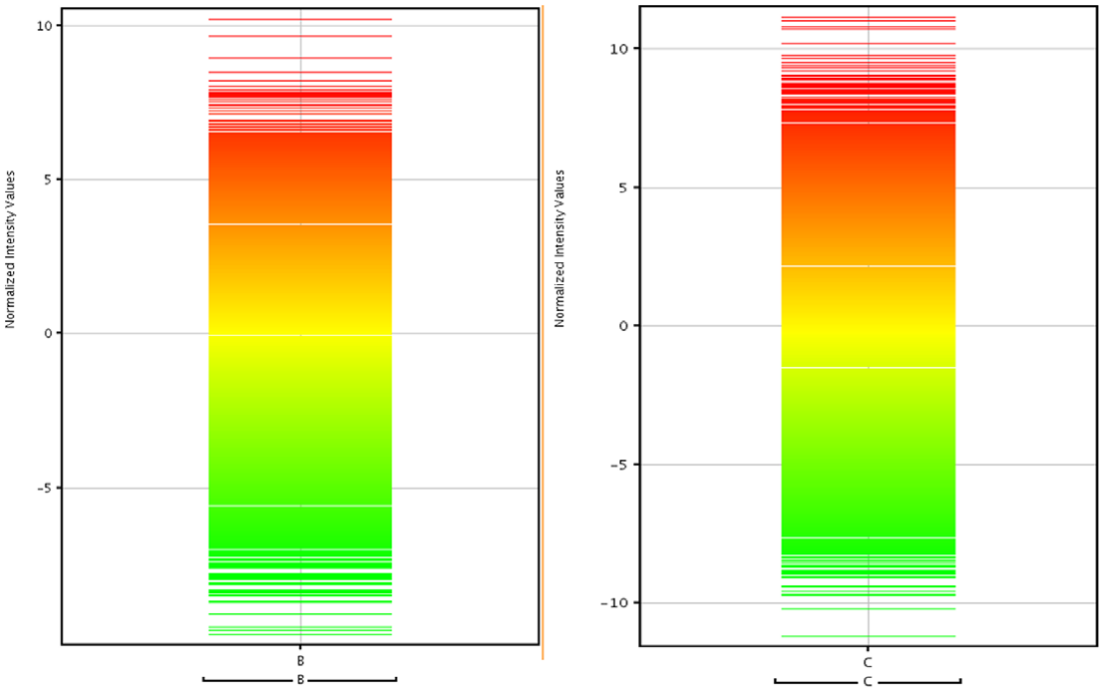
Expression profile of the differentially expressed transcripts (A) in cultivar B with respect to A (B) in cultivar C with respect to A.

In this study, cultivar Nattu was used as control and the differential expression of transcripts in Erode and Mysore cultivars were determined. We observed 1,774 and 1,356 differentially expressed transcripts in Erode and Mysore cultivars respectively (Figure 6). Of the 1,774 differentially expressed transcripts in cultivar Erode, we observed 629 upregulated transcripts and 1,145 downregulated transcripts. Similarly, in cultivar Mysore, we found 793 upregulated transcripts and 563 downregulated transcripts. We were not able to find significant expression level differences among the three cultivars at the transcriptome level. However, one significant observation in the expression analysis is that hypericin, a secondary metabolite with antitumor [23], [24] and antibiotic properties [25], was found to be upregulated in both Erode and Mysore cultivars.

### Identification of Simple Sequence Repeats (SSRs)

10,108 SSRs in 8,315 sequences exceeding 12 bases were recognized in cultivar Nattu (Additional file S8). Cultivar Erode showed 11,957 SSRs in 9,851 sequences (Additional file S9) and cultivar Mysore showed 24,987 SSRs in 18,955 sequences (Additional file S10). Trinucleotide repeats were the most frequent SSR motifs followed by tetranucleotide, dinucleotide, pentanucleotide and hexanucleotide motifs (Table 5).

**Table 5:** Summary of SSRs.

Motif size	SSRs observed in Cultivar Nattu	SSRs observed in Cultivar Erode	SSRs observed in Cultivar Mysore
2	1014 (10%)	1364 (11.4)	3023 (12.1%)
3	5646 (55.9%)	6421 (53.7%)	12927 (51.7%)
4	2150 (21.3%)	2707 (22.7%)	5973 (23.9%)
5	550 (5.4%)	683 (5.7%)	1463 (5.9%)
6	748 (7.4%)	782 (6.5%)	1600 (6.4%)

## Discussion

Next Generation Sequencing technologies have revolutionized sequencing and sequencing is no longer laborious and cost-intensive. Transcriptome sequencing of non-model plants are gaining importance in recent years as it allows sequencing only the transcribed regions at low cost.

We obtained more than 90% HQ bases (Table 1) for all the cultivars which reflects a high quality sequencing run. Low quality bases as well as the presence of adapters in reads could interfere with the assembly process resulting in misassemblies or truncated contigs. Hence, low quality bases and adapter sequences were trimmed before proceeding with further analysis. Such trimmed HQ reads were used to arrive at a high quality assembly. N50 statistic is widely used to assess the quality of the assembly. Higher the N50 value better the assembly is. The observed N50 value (1,515 bases) was higher than those obtained in other plant transcriptome sequencing projects (Table 6) suggesting a better assembly. Nonetheless, a better assembly does not guarantee an accurate assembly because assembling plant sequences pose different challenges, as plants can have higher rates of heterozygosity and repeats [26]. However, with improvements in assembly algorithms [27], accurate assemblies will be made possible in future. Clustering of the three assemblies is expected to reduce the sequence redundancy arising either out of merging three assemblies or multiple isoforms inherent to an individual assembly or both. Clustering also enriches the information contained in the cluster by complementing from all three assemblies.

**Table 6:** Comparative analysis of Plant transcriptome N50 values.

Organism	N50 (in bases)
*Acacia auriculiformis* [17]	948
*Acacia mangium* [17]	938
*Daucus carota* var. sativus L. [12]	1378
*Cicer arietinum* L. [11]	1192
*Euphorbia fischeriana* [19]	∼1500
*Cajanus cajan* L. [18]	1510
*Hevea brasiliensis* [13]	485
*Sesamum indicum* L. - 3 libraries [14]	220, 150, 180
*Ipomoea batatas* [15]	765
*Camellia sinensis* [16]	506

COG and GO annotations indicate the presence of transcripts involved in stress resistance and defence mechanisms. In this study we have included a pathway annotation even if only a single enzyme from a pathway is observed. For example, out of twenty different enzymes involved in perillyl alcohol biosynthesis we observed only (−)-limonene-3-hydroxylase enzyme once, yet there is phytochemical evidence for its presence in turmeric [28]. This justifies our decision to include all pathway annotations without setting any threshold. Sequence similarity search indicated the presence of curcumin synthase, an enzyme involved in the synthesis of curcumin, well known as a potent anti-cancer compound. It is known to act at various chronological stages, right from initial insults that cause DNA damage to metastasis by modulating a multitude of pathways. Effects of curcumin on cell cycle regulation, apoptosis, NF-κB and AP-1 transcription factors, autophagy, angiogenesis and metastasis have been well elucidated [3], [29]–[37].

**Figure 7:**
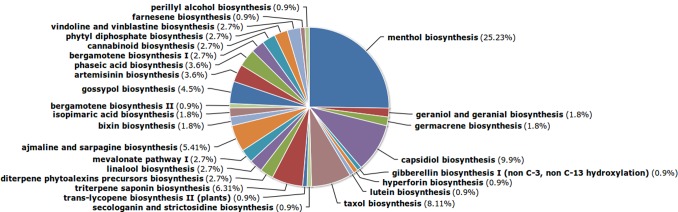
Terpenoid pathways represented in the PlantCyc annotation of the representative transcripts.

It has been suggested that plants have developed terpene based host defence, which also represents a repertoire of therapeutic compounds [38]. Hence in this study, we have focused our analysis towards terpenoids. Major share of transcripts related to terpenoid pathways were found to be from menthol biosynthesis (25%). Recent evidences indicate that menthol has a potent anticancer property, effecting cell death through TRPM8 receptor [39]. Here in this study for the first time, we report the present of genes related to taxol biosynthetic pathway in turmeric. Taxol biosynthetic pathway was the third most represented (8.11%) terpenoid pathway in the transcriptome (Figure 7). Further studies into this pathway in turmeric could be benificial as the combination of taxol with curcumin has produced promising results in treating cancer [40].

We also observed transcripts related to pathways involved in synthesis of several other anti-cancer compounds including vincristine, vinblastine, matairesinol, hypericin, xanthohumol, simplecoumarins, geraniol and coumestrol [23], [41]–[47]. Epidemiological studies suggest that lower incidences of colon cancer in India is due to consumption of diets rich in curcumin [48], but the presence of transcripts related to biosynthetic pathways of other anti-cancer molecules suggest that they along with curcumin might have elicited an effect in preventing cancer. However, further studies in this direction will help in validating this assertion.

Pathway annotation of transcripts showed the presence of transcripts related to artemisinin and acridone alkaloids biosynthetic pathways. Both artemisinin and acridone alkaloids are proven to be effective in anti-malarial treatments[49]–[50]. Moreover curcumin *per se* has been reported to have anti-malarial activity as it is known to exhibit prooxidant properties in *P. falciparum,* at concentrations which are non-toxic to mammalian cells, inducing oxidative damage resulting in the death of parasite [51].

Our analysis revealed a number of transcripts of such sesquiterpenes as capsidiol, gossypol, phaseic acid, bergamotene, germacrene and farnesene when compared to monoterpenes linalool, geraniol, menthol and perillyl alcohol. It has been demonstrated that essential oils from leaves are usually dominated by monoterpenes while the oils from rhizomes mainly contains sesquiterpenes, which are synthesized in response to a pathogenic attack [28]. Our findings are consistent with this statement.

SNPs and Polymorphic SSR markers play an important role in genetic diversity analysis. The influence of SSRs on gene regulation, transcription and protein function typically depends on the number of the repeating units [52]. In the present study, trinucleotide type SSR motifs occurred more frequently which is consistent with findings from other studies involving monocots, like rice, barley and wheat [53].

The *de novo* transcriptome of this very important phytochemical herb brings out for the first time novel transcripts related to anticancer, antimalarial and anti-oxidant properties. Proper validation of the results at biochemical, cellular and animal model studies will certainly highlight more useful properties of turmeric in traditional and alternative medicine. The data may also aid plant breeders to engineer cultivars with enhanced terpenoid profiles.

## Methods

### Sample Collection and Preparation

Rhizome samples of the following three widely grown cultivars in South India were chosen for our study, cultivar Nattu, cultivar Erode and cultivar Mysore. RNA was extracted from the rhizome samples frozen in liquid nitrogen, using Agilent Plant RNA isolation mini kit (Product No; 5188–2780) and was quantified using Nanodrop. QC was performed using Agilent's Bioanalyzer. RNA Integrity Number (RIN) was observed to be 7.6, 7 and 8.3 for cultivars Nattu, Erode and Mysore respectively. Transcriptome library for sequencing was constructed as outlined in Illumina's “TruSeq RNA Sample Preparation Guide” (Part # 15008136; Rev. A; Nov 2010).

### Sequencing and Quality Control

Illumina GAIIx was used to generate 72 (Cultivar Nattu), 73 (Cultivar Erode) and 100 (Cultivar Mysore) base paired-end short reads using Sequencing By Synthesis (SBS). Standard Illumina pipeline (RTA-CASAVA-OLB) was used to generate short reads in FASTQ format. Accuracy of base calling is reflected in the quality scores and low quality scores usually denote high error probabilities. Low quality bases, if due to errors, will interfere in the assembly process either resulting in misassemblies by collapsing repeat regions or truncated contigs by obscuring true overlaps [26]. Hence, quality filtering is very essential in order to arrive at a high quality assembly. Hence additional quality control was performed using in-house program (SeqQC V2.1 - http://genotypic.co.in/SeqQC.html) to generate high quality reads for use in assembly. The reads were filtered or trimmed for adapters, B trimming (CASAVA1.7 User Guide) and other low quality bases using in-house Perl scripts. These high quality, filtered reads were used for further analysis.

### Transcriptome Assembly and Clustering

Contig assembly for all the three cultivars was carried out using a de Bruijn graph based *de novo* genome assembler Velvet-1.1.07 (http://www.ebi.ac.uk/~zerbino/velvet/) [54]. Velvet takes in short reads and assembles them into contigs using paired-end information. A draft assembly was built with following parameters: hash length = 45, expected coverage = auto and coverage cutoff = auto. This draft assembly was used by observed-insert-length.pl and estimate-exp_cov.pl (from Velvet package) to estimate insert length and expected coverage parameters, which were then used to generate a final assembly. The values of the estimated insert length, insert length standard deviation and expected coverage for the three draft assemblies are as follows, Cultivar Nattu: 153, 53.17, 5; Cultivar Erode: 151, 49.67, 5 and Cultivar Mysore: 150, 44.55, 3. The resulting contigs were assembled into transcripts by Oases-0.2.01 (http://www.ebi.ac.uk/~zerbino/oases/) [55], which uses the assembly from Velvet and clusters them into small groups (loci). It then uses paired end information to construct transcript isoforms. Assembly statistics were calculated using in-house Perl scripts.

The transcripts from three individual assemblies were clustered (CD-HIT v4.5.4 http://www.bioinformatics.org/cd-hit/) [56] in order to generate a comprehensive reference. Sequence identity threshold and alignment coverage (for the shorter sequence) were both set as 80% to generate clusters. Such clustered transcripts were defined as reference transcripts in this work.

### Functional Annotation

#### Database

No single database could be used to comprehensively annotate the transcripts. Hence using multiple databases for annotation could help in rich annotation of the transcripts and thereby providing insights into the function. In this study we have used six databases to derive annotation, which include Viridiplantae mRNA dataset from NCBI's GenBank (3,184,383 sequences as of 14^th^ March 2012), Swiss-Prot (34,371 sequences as of 21^st^ March 2012) [57], TrEMBL (1,127,879 sequences as of 21^st^ March 2012) [57], PlantCyc Enzymes database (v2.0) [58], KOG proteins from Clusters of Orthologous Groups (COG) database (112,920 sequences) [59] and Pfam (v26.0) [60].

#### BLAST search

Sequence homology search was performed (BLAST v2.2.25+ http://blast.ncbi.nlm.nih.gov/Blast.cgi) at an E-value cut-off of e-5 (<0.00001) [61]. Megablast search was performed against Viridiplantae database while blastx search was carried out against Swiss-Prot, TrEMBL, KOG and PlantCyc Enzymes. BLAST annotations were filtered using either subject or query coverage (>30%) and sequence identity (>50% for megablast and identity >30% for blastx). InterProScan-4.8 (http://www.ebi.ac.uk/Tools/pfa/iprscan/) was used to scan Pfam database for identifying protein domains [62].

To make a final annotation table, a transcript's best annotation was chosen based on the BLAST scores [61]. Swiss-Prot, PlantCyc and KOG databases were given preferences and if a transcript does not have annotation in these databases then either GenBank Viridiplantae mRNA or TrEMBL annotation was chosen based on the blast scores and if a transcript does not have annotations in any of the above databases, Pfam annotation is assigned to the transcript.

### BLAST against Curcuma Longa Nucleotide Sequences and ESTs from ArREST

As of 19^th^ April 2012, 12,833 Curcuma longa sequences (240 nucleotides and 12,593 ESTs) were available NCBI and were downloaded for BLAST search against representative transcripts at an E-value threshold of e-5 (<0.00001). Although, PlantCyc database (v2.0) did not contain curcumin biosynthetic pathway, all 3 isoforms of curcumin synthase mRNA (AB495007.1, AB506762.1 and AB506763.1) were available in NCBI. Hence, this BLAST search was also used to confirm the presence of curcumin synthase.

A total of 78,516 *C. longa* ESTs were downloaded from Aromatic Rhizome EST (ArREST) database http://www.plantrhizome.org/download/. All of these sequences were used for BLAST search against RT using an E-value threshold of e-5 (<0.00001).

### Mapping, Calling Variations and Quantifying Transcripts

High quality filtered reads from each Cultivar were individually aligned (bowtie2 v2.0.0-beta5 http://bowtie-bio.sourceforge.net/bowtie2/index.shtml) [63] to the RTs. End-to-end alignment allowing non-discordant read alignment was performed, with insert size of 900 bases, and only the best alignment were reported. The alignment was generated in Sequence Alignment/Map format. The alignments were processed for further analysis like variant calling using SAMtools v0.1.7a (http://samtools.sourceforge.net/) [64]. A combination of reads showing variation and read depth, along with mapping quality and SNP quality were considered for filtering the SNPs (Additional file S11).

Differential expression analysis was performed by employing a negative binomial distribution model (DESeq v1.8.1 package http://www-huber.embl.de/users/anders/DESeq/) [65]. Dispersion values were estimated with the following parameters: method = blind, sharingMode = fit-only and fittype = local. Cultivar Nattu was considered as a control to compare against other two cultivars (Erode and Mysore). P value threshold of 0.01 was used to filter statistically significant results.

### Identification of SSRs

SSRs were detected using MIcroSAtellite Identification Tool (MISA v1.0). Minimum unit size cut-off of 6 was used to report a dinucleotide repeat, 4 for a trinucleotide repeat and 3 for SSRs of sizes 4, 5 and 6. A maximum distance of 100 nucleotides was allowed between two SSRs.

## Supporting Information

File S1 **BLAST against** ***Curcuma longa*** **nucleotide sequences.** The table lists the best hit for each RT, with similarity to *Curcuma longa* ESTs.
(XLS)

File S2 **BLAST against ESTs from ArREST.** The table lists the hits for each RT, with similarity to ArREST ESTs.
(ODS)

File S3 **Swiss-Prot table.** The table lists the Swiss-Prot annotations from which Gene Ontology terms were determined.
(XLS)

File S4 **Pathway table.** The table lists the enzyme annotations from which pathway were determined.
(XLS)

File S5 **KOG table.** The table lists the KOG annotations from which KOG terms were determined.
(XLS)

File S6 **Pfam table.** The table lists the pfam annotations from which domains were determined.
(XLS)

File S7 **Final table.** The table lists the best annotation for each transcript.
(XLS)

File S8 **SSR information Cultivar A.** This file provides SSR information for sample A.
(XLS)

File S9 **SSR information Cultivar B.** This file provides SSR information for sample B
(XLS)

File S10 **SSR information Cultivar C.** This file provides SSR information for sample C
(XLS)

File S11 **SNP filtering criteria.** The file provides criteria used for filtering SNPs.
(DOC)
